# The Effects of Oil Extraction Methods on Recovery Yield and Emulsifying Properties of Proteins from Rapeseed Meal and Press Cake

**DOI:** 10.3390/foods9010019

**Published:** 2019-12-24

**Authors:** Karolina Östbring, Emma Malmqvist, Kajsa Nilsson, Ia Rosenlind, Marilyn Rayner

**Affiliations:** Department of Food Technology, Engineering and Nutrition, Lund University, 221 00 Lund, Sweden; emmamalmqvist@gmail.com (E.M.); kajsa.nilsson@food.lth.se (K.N.); ia.rosenlind@food.lth.se (I.R.); marilyn.rayner@food.lth.se (M.R.)

**Keywords:** rapeseed press cake, plant proteins, protein recovery, emulsifying properties

## Abstract

The agricultural sector is thought to be responsible for around 30% of the anthropogenic climate change and it is well established that high meat consumption has a tremendous impact on the environment. Rapeseed is mainly used for production of vegetable oil, but press cake has high protein content with the potential for incorporation into new plant protein-based foods. Protein was recovered from press cakes generated from different oil pressing processes. Industrially cold-pressed, hot-pressed, and solvent-extracted rapeseed press cake and the effect of heat treatment in the recovery process was assessed. Protein recovery yield, protein concentration and emulsifying properties were analyzed. Cold-pressed rapeseed press cake (RPC) recovered in the absence of heat, yielded the highest protein recovery (45%) followed by hot-pressed rapeseed meal (RM) (26%) and solvent-extracted RM (5%). Exposure to heat during recovery significantly reduced the yield for cold-pressed RPC but no difference was found for hot-pressed RM. The protein recovery yield was improved for solvent-extracted RM when heat was applied in the recovery process. The ability to stabilize emulsions was highest for protein recovered from cold-pressed RPC, followed by hot-pressed RM and solvent-extracted RM, and was in the same range as commercial emulsifying agents. Heat treatment during recovery significantly reduced the emulsifying properties for all pressing methods examined. This study suggests that cold-pressed rapeseed press cake without heat in the recovery process could be a successful strategy for an efficient recovery of rapeseed protein with good emulsifying properties.

## 1. Introduction

A robust body of research indicates that the dietary choices of modern society with high levels of meat consumption, have tremendous impacts on the environment [1]. Around 30% of the anthropogenic climate change is linked to the agricultural sector [2]. Protein from animal sources has a significantly higher global warming potential as compared with plant-based protein [3,4]. Ruminants are associated with significantly higher levels of greenhouse emissions than monogastric animals, explained mainly by the methane emissions from enteric fermentation [5,6]. A change to diets that include more plant-based proteins is a potential area for mitigating the agricultural sectors emissions. Such a change in consumption patterns creates a need for new plant-based foods rich in dietary protein [7].

Rapeseed (*Brassica napus*, *Brassica rapa,* and *Brassica juncea* of rapeseed quality) is the world’s second largest cultivated oilseed and, in 2017, a total of 76.2 million tons were produced by 63 countries around the world [8]. Rapeseed is mainly used for production of vegetable oil [9] but the residual press cake has a high protein content. For every kilogram of rapeseed oil, double the amount (two kilograms) of press cake is generated, and the press cake or meal is predominantly used as a protein source in feed for dairy cattle, poultry, and pigs, as well as limited use in aquaculture [10]. Rapeseed protein is comparable with soy protein in nutritional value and contains a higher amount of S-amino acids than many other plant proteins [11].

However, the utilization of rapeseed protein as human food is limited due to the presence of antinutritional compounds such as glucosinolates, phytates, and phenols [12,13]. Glucosinolates are not harmful in their original state but can be hydrolyzed to glucose and a range of potentially toxic chemicals such as isothiocyanate or thiocyanate ions by the enzyme myrosinase when the rapeseed is crushed and enzymes are liberated. The intestinal tract microflora can also metabolize glucosinolate [11]. Toxic effects of glucosinolate breakdown products in animals include reduced feed intake, growth depression, impaired thyroid function, and enlargement of the liver [11]. Due to the harmful effects of glucosinolates in rapeseed, the plant breeding field has focused on reducing the initial concentrations at the seed level and has successfully reduced the levels by a factor of ten since the 1970s. However, glucosinolates are water soluble and are concentrated in press cake after oil extraction of the rapeseed. Phytic acid is a strong chelating agent that can bind metal ions, especially zinc and iron, and thereby affect the uptake of minerals from other ingested foods which can cause a problem with mineral balance in the diet [14]). Phenolic compounds contribute to a bitter flavor and have a dark greyish color and levels should be reduced to allow formulation of a palatable food product [11]. Recently, the field of industrial filtration has successfully created filters with higher capacity and lower price [15], and filtration has been reported to successfully reduce or eliminate antinutrients from the rapeseed protein dispersion [14,16]. Therefore, rapeseed protein has the potential to serve as an alternative plant protein source for human consumption due to its high protein quality and good techno-functional properties [17,18,19].

Most of the literature on extraction of rapeseed proteins for human consumption has focused on hot-pressed or solvent-extracted rapeseed meal and less attention has been given to industrially cold-pressed rapeseed as a potential raw material for new plant-based foods [20,21,22,23,24]. Very briefly, there are mainly two strategies to produce rapeseed oil, i.e., cold press and hot press. For production of cold-pressed rapeseed oil, the seeds are cleaned, and the moisture content is adjusted to 7% to 8% followed by a mechanical pressing where the oil temperature is kept below 40 °C. Besides the oil, a press rest (i.e., the press cake) is generated with an oil concentration of 16% to 20%. The oil is filtered to remove fine solids followed by packaging. For production of hot-pressed rapeseed oil, the seeds are cleaned, and the moisture content is adjusted. Thereafter, the seeds are crushed or flaked followed by a cooking step (80 °C to 100 °C) to rupture the cell walls, and thereby facilitate the later separation of oil and dry solids. The crushed seeds are pressed, and the press rest, named rapeseed meal, when hot pressed, contains approximately 4% oil. The meal can be treated with solvents (i.e, hexane) to further extract oil using a process which includes boiling at 107 °C for approximately 1 h, and thereby increases the oil yield. The solvent-extracted rapeseed meal has an oil concentration of ≤1%. For hot-pressed rapeseed oil production, there are several additional process steps after the pressing and solvent treatment to refine the oil and prolong the shelf life [10].

The dominating protein groups in rapeseed are the storage proteins napin (albumin) and cruciferin (globulin) which together constitute 85% to 90% of the total proteins. The oil body proteins, oleosin and caleosin, account for the remaining part [25]. Several different isoelectric points can be found in the literature and the complex protein matrix could be the reason. Most frequently, napin has been reported to have an isoelectric point of 10.5, cruciferin 7.25, and oleosin 6.5 [16]. A mixture of proteins in rapeseed protein concentrate has lower isoelectric points as compared with purified protein groups, differing between a pH of 3.5 to 6. It has been proposed that the botanical variety is important for the isoelectric point of the rapeseed proteins [19].

If rapeseed proteins are to be formulated to food products, not only the protein recovery yield, but also the techno-functional properties such as the ability to stabilize emulsions need to be investigated and optimized. Emulsions are a mixture of two immiscible phases, i.e., oil and water where one is dispersed into the other in the form of small droplets and these droplets need to be stabilized by an emulsifier to prevent coalescence. Many of our most popular foods are in emulsion form, such as salad dressings, mayonnaise, ice cream, beverages, and sauces. The majority of the natural protein-based emulsifiers in the food industry has its origin in foods such as milk, egg, or soy. The food industry has a need for plant-based protein with low-cost emulsifying capacity for use in plant-based food applications, and rapeseed protein has been reported to have good emulsifying properties, and therefore could be an interesting candidate [26,27]. The high emulsifying capacity is related to the protein structure in rapeseed.

Oil is stored in oilseeds in the form of oil bodies surrounded by both a monolayer of phospholipids, as well as oil body proteins to stabilize the oil–water interface. These oil bodies occur as individual entities and do not aggregate or coalesce even when subjected to environmental stress such as draught [26]. The arrangement with oil stored in oil bodies, increases the surface area and allows rapid access to energy when the environment is favorable for growth. The stability of oil bodies is attributed to oleosins’ unique structure. The N-terminal is folded to expose both hydrophilic and hydrophobic regions where the hydrophilic parts are facing the aqueous solution and the hydrophobic parts are facing the lipid phase. Oil body proteins also have a long hydrophobic region and the folding allows a 180 °C turn, anchoring the protein into the oil droplet [28]. Oil body proteins are designed to protect oil droplets from coalescence and these inherent properties can also be used to stabilize food emulsions [29]. Of the storage proteins, cruciferin exhibits better emulsifying properties as compared with napin [30]. The protein recovery yield and techno-functional properties of the protein depend largely on the quality of the starting material and the oil extraction method used, since functionality mcan vary depending on processing strategies [11].

The presence of antinutritional components in rapeseed press cake or meal requires extraction of the proteins in order to formulate safe food products for human consumption. During protein extraction, the amount of antinutrients is reduced due to dilution during washing. Adding value to the by-streams is of great economic importance for oil producers, and efforts to recover protein from those streams is one strategy to increase the sustainability of the oil sector. To our knowledge, there are no studies evaluating the effect of the pressing method and exposure to heat on the protein recovery yield and emulsifying properties of large-scale industrial by-streams of rapeseed press cake and meal. The aim of this study was, therefore, to investigate the following: (i) To what extent exposure to heat and solvents during the oil pressing and (ii) to what extent exposure to heat, during the protein extraction process, affects the protein recovery yield and the proteins’ emulsifying properties for industrial cold-pressed, hot-pressed, and solvent-extracted rapeseed.

## 2. Materials and Methods

### 2.1. Materials and Chemicals

Cold-pressed rapeseed press cake (RPC) (*Brassica napus* L.) was a kind gift from Gunnarshögs Jordbruk AB (Hammenhög, Sweden). The rapeseeds were cold-pressed at Gunnarshögs Jordbruk AB’s production site and the oil temperature during pressing did not exceed 35 °C and no solvents were used. The hot-pressed rapeseed meal (RM) and the solvent-extracted RM was a kind gift from AAK AB (Karlshamn, Sweden). Hot pressing was typically performed at 80 °C to 100 °C. For the solvent-extracted meal, the oil residues were recovered by hexane post hot pressing. Citric acid (C_6_H_8_O_7_, CAS 77-92-9), sodium chloride (NaCl, CAS 7647-14-5), sodium dihydrogen phosphate monohydrate (H_2_Na PO_4_·H_2_O, CAS 7558-80-7), disodium hydrogen phosphate dodecahydrate (Na_2_HPO_4_·12H_2_O, CAS 10039-32-4), and sodium hydroxide (NaOH, CAS 1310-73-2) were purchased from Merck (Darmstadt, Germany). Miglyol 812 was purchased from Sasol AG (Witten, Germany). Bovine serum albumin (BSA) and chicken egg white albumin were purchased from Sigma-Aldrich (St. Louis, MA, USA) and deoiled lecithin from soy was purchased from Cargill (Minneapolis, MN, USA). All other chemicals were of analytical grade.

### 2.2. Protein Extraction from Rapeseed Press Cake

**Figure 1:**
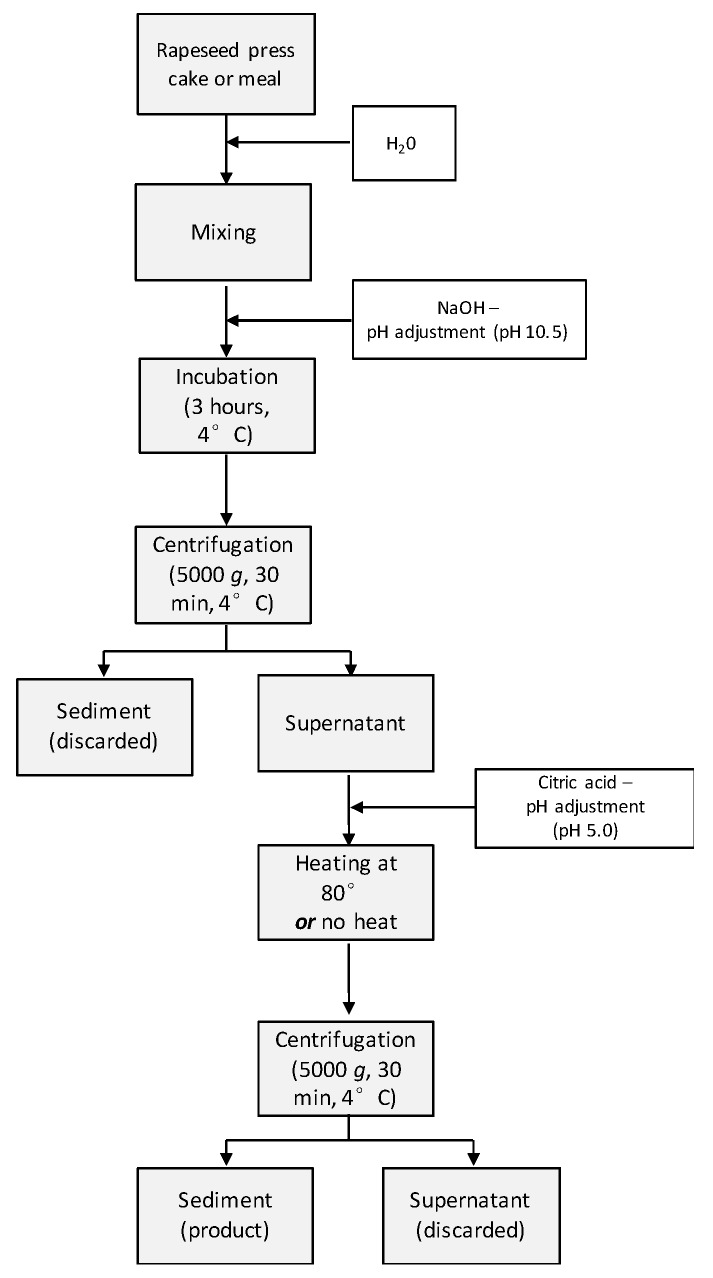
Schematic illustration of the extraction process of rapeseed protein from press cake or meal to precipitated protein extract.

Protein was recovered from cold-pressed RPC, hot-pressed RM, and solvent-extracted RM (Figure 1). The protein recovery procedure was identical for all raw materials used and was modified from the procedure previously described by Wijesundera [31]. The rapeseed press cake or meal was stored in a freezer (−18 °C) until the start of the protein recovery processes. The rapeseed press cake or meal (50 g) was ground in a knife mill (Grindomix GM 200, Retsch, Germany) for a total of 20 s in 4 s intervals. The pulverized press cake was hydrated in tap water (1:10 *w*/*w*) and the pH was adjusted to 10.5 with 2M NaOH. The dispersion was mixed for 10 min (IKA Labortechnik, Eurostar digital) at 160 rpm, the pH was readjusted to 10.5, and the dispersion was incubated in 4 °C for 3 h. After incubation, the dispersion was centrifuged (Beckman Coulter, Allegra® X-15R Centrifuge, Brea, CA, USA) for 30 min at 5000× *g* in 4 °C. The supernatant was collected, and the pH was adjusted to 5.0 with citric acid powder. Half of the dispersion experimental set was heat treated at 80 °C on an induction stove. The start temperature of the dispersions was 19 ± 1 °C, the come-up time was 227 ± 6 s, and the holding time at 80 °C was 3 s. Then, the dispersions were immediately cooled in a cold-water bath until 25 °C and the cooling time was 333 ± 7 s. The other half set was not heat treated. The dispersions were again centrifuged (Beckman Coulter, Allegra® X-15R Centrifuge, Brea, CA, USA) for 30 min at 5000× *g* in 4 °C and the sediment was collected. The sediment containing rapeseed protein precipitate was used for all other experiments and was stored in a freezer (−18 °C) until further analysis. Three batches were prepared for each raw material with and without heat treatment in the recovery process.

### 2.3. Proximate Analysis

Protein content was analyzed using the Dumas method for both the rapeseed press cake from each raw material, as well as for the subsequent protein precipitate (sediment in the last step of the extraction process). Nitrogen content was determined by the elemental analyzer Flash EA 1112 (Thermo Electron Co., Waltham, MA, USA) blanked with air and with aspartic acid as a reference. Approximately 25 mg material was placed in a tin cylinder (diameter 30 mm) for analysis. A conversion factor of 6.25 was used to calculate the protein content. Each extraction batch was analyzed in triplicate (nine measurements per processing method with and without heat treatment during protein recovery). The protein recovery yield was calculated as follows:

(1) $$\begin{array}{cc} \mathrm{Protein}\;\mathrm{recovery}\;\mathrm{yield}\;\left(\%\right) & \frac{\mathrm{Protein}\;\mathrm{amount}\;\mathrm{in}\;\mathrm{rapeseed}\;\mathrm{protein}\;\mathrm{precipitate}\;\left(g\right)}{\mathrm{Protein}\;\mathrm{amount}\;\mathrm{in}\;\mathrm{press}\;\mathrm{cake}\;\mathrm{or}\;\mathrm{meal}\left(g\right)\;}\times 100\% \end{array}$$

The moisture content of the precipitates was determined according to the official method of analysis (AOAC 2007). The temperature was adjusted to 102 °C and analyses were performed in triplicate. Determination of the oil content in rapeseed sediments by solvent extraction was performed in at least duplicate in a semiautomatic Soxtec apparatus (Tecator AB, Höganäs, Sweden) using petroleum ether as a solvent. Oil content was expressed in a dry-matter basis.

### 2.4. Emulsifying Properties of Emulsions Stabilized by Rapeseed Proteins

Emulsifying properties were investigated for rapeseed protein precipitates from cold-pressed RPC, hot-pressed RM, and solvent-extracted RM with and without heat in the recovery process.

#### 2.4.1. Preparation of Emulsions

Oil-in-water emulsions were prepared in triplicate in glass test tubes with 2 mL continuous phase (0.005 M phosphate buffer, 0.2 M NaCl, pH 7), 1 mL dispersed phase (Miglyol 812), and varying amounts of precipitated rapeseed protein extract. Concentrations of 2, 4, 8, 16, and 32 mg rapeseed protein/mL oil of each rapeseed protein precipitate were made. The emulsions were homogenized with a mixer (Ystral D-79282, Ballrechten-Dottingen, Germany) at 22,000 rpm for 60 s. Then, the emulsions were incubated for 1 h at 4 °C, prior to particle size analysis.

In order to compare the emulsifying capacity for rapeseed proteins with commercial emulsifiers, emulsions were prepared as described above with a phosphate buffer, miglyol oil, and varying amounts of BSA, with deoiled soy lecithin and egg white albumin as emulsifiers.

#### 2.4.2. Particle Size Analysis of Emulsions

Particle size distribution of emulsions stabilized by rapeseed protein were analyzed with a laser diffraction particle analyzer (Mastersizer 2000 Ver 5.60, Malvern, Worcestershire, UK). The pump velocity was 2000 rpm with 100 mL MilliQ^®^ water in the sampling chamber. The test tubes were tilted three times to get a homogenous sampling. Each emulsion replicate was measured three times and the average was reported. The refractive index (RI) was 1.45 for the miglyol oil and 1.33 for the water. The obscuration was between 10% to 20%. From the particle size distribution, the volume weighted mean *d*_43_, mode (top of the peak), and volume (%) were calculated.

### 2.5. Statistical Analysis

All analyses were carried out in triplicate and possible differences between the means were analyzed using Student’s *t*-test. Results were considered significant if *p* values were ≤0.05. All results were expressed as means with standard deviation.

## 3. Results and Discussion

### 3.1. Effect of Pressing Process on Protein Recovery Yield

**Figure 2:**
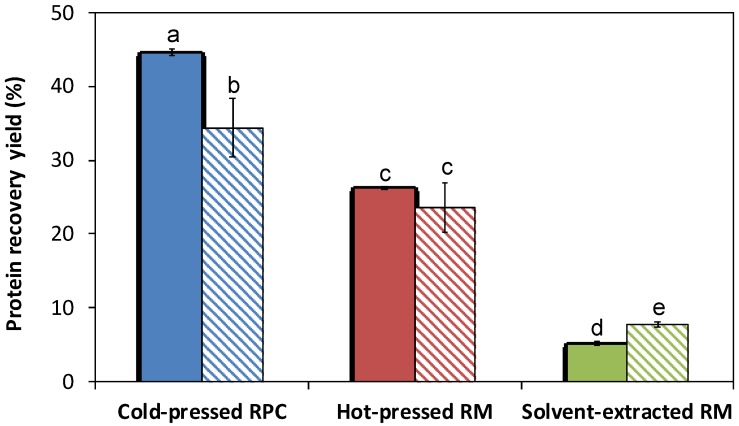
Protein yield for rapeseed recovered from cold-pressed RPC, hot-pressed RM, and solvent-extracted RM. Solid bars are protein recovery yield without heat in the recovery process and striped bars are protein yield recovered with heat. Different letters indicate significant difference (*p* ≤ 0.05). RPC = rapeseed press cake, RM = rapeseed meal.

The protein recovery yield was significantly higher for cold-pressed RPC (45 ± 0.1%) as compared with hot-pressed RM (26 ± 0.2%, *p* ≤ 0.001) and solvent-extracted RM (5.1 ± 0.3%, *p* ≤ 0.001) (solid bars in Figure 2). Furthermore, hot-pressed RM had a significantly higher protein recovery yield as compared with solvent-extracted RM (*p* ≤ 0.001). Proteins, in general, are heat sensitive and, in a study by Wu and Muir, a rapeseed protein isolate was reported to have a denaturation onset temperature of 77.9 °C. When proteins were investigated by group, the temperature for onset of denaturation was higher and cruciferin started to denature at 82.2 °C, whereas napin was more heat stable with a denaturation onset temperature of 96.8 °C [32]. Hot-pressed RM was exposed to temperature above the onset of denaturation, but the cold-pressed RPC exited the industrial screw press at 50 °C to 55 °C depending on the outdoor temperature. Our hypothesis is that the proteins in the hot-pressed samples were partly denatured by heat [11] during the pressing and could not be solubilized by the alkali pH during the extraction process to the same extent as proteins in the cold-pressed RPC. The extensive heat treatment (1 h at 107 °C) for the solvent-treated RM reduced the protein recovery even further as compared with hot-pressed starting material. Hence, the highest protein recovery yield was achieved from extractions from cold-pressed RPC. Similar results were reported by Fetzer et al. where a higher proportion of the rapeseed proteins could be solubilized in the alkali extraction if cold-pressed RPC was used (52.3%) as compared with hot-pressed RM (36.7%) [23]. However, Fetzer et al. investigated rapeseed protein for non-food applications and focused solely on soluble proteins, and therefore no precipitation step was included in their study design. The extraction yield in the study by Fetzer et al. should not be compared to the protein recovery yield in our study in absolute terms. However, this study supports the results presented by Fetzer et al. where a higher yield of proteins were extracted from cold-pressed as compared with hot-pressed rapeseed.

Heat is often used as a strategy to separate proteins in aqueous solutions by precipitation [33], but in our study, exposure to heat in the protein recovery process (80 °C for a few seconds) yielded reduced protein recovery yield for cold-pressed RPC (*p* ≤ 0.05) although the difference was not significant for hot-pressed RM (*p* = 0.37) (striped bars in Figure 2). For solvent-extracted RM, the protein recovery yield was instead slightly increased (*p* ≤ 0.05) when exposed to heat. The onset of denaturation has been reported to be 77.9 °C for rapeseed protein isolate with a denaturation temperature of 83.9 °C [32]. However, the rapeseed proteins in our study had already coagulated at 72 °C to 74 °C for all pressing methods investigated. Wu and Muir investigated denaturation temperature by differential scanning calorimetry where a sample was heated rapidly. In our study the come-up time was around 3.5 m which could have affected the results. The protein composition in rapeseed is also very complex and varies between botanical varieties and the varieties used in our study seem to have an overall lower denaturation temperature as compared with Wu and Muir. The effect of heat in the recovery process had less effect on the protein yield as compared with the pressing method.

The protein recovery yield in our study is in agreement with values reported previously, although no studies have been conducted specifically on precipitated proteins from industrially cold-pressed RPC. Tan et al. supported our finding that the protein yield was higher for non-toasted RPC as compared with industrially toasted rapeseed meal [27]. However, the protein recovery yield for non-toasted RPC reported by Tan et al. was significantly lower (15%) than the yield reported in our study (45%). Tzeng et al. found the protein recovery yield to be 37.8% for commercial hot-pressed RM [14], although several additional steps were included in the referred study resulting in a protein isolate with higher purity.

It is well-known that increased pH in the extraction phase is associated with higher protein recovery yields, with a pH of 12 yielding the highest protein recovery [31,34,35]. However, it is neither industrially nor environmentally feasible to use a pH of 12 in the extraction process, and therefore a pH of 10.5 was used in our study as a compromise between high protein recovery yield and respect for environmental factors. Chabanon et al. reported the protein recovery yield to be 30% in a study where hot-pressed RM was mixed with a water/ethanol mix, and the pH was adjusted to 4.5 [36]. Both isoelectric precipitated proteins (globulins) and soluble proteins (albumins) were included in the reported yield. In our study, both isoelectric pH (5.0) and heat (80 °C) were used to precipitate the proteins, hence both globulins and albumins were expected to be present in the final protein sediment. The higher protein recovery yield in the study by Chabanon et al. could be attributed to different pretreatments of the rapeseed meal. Blaicher et al. [34] found the protein yield to be as high as 49% when hot-pressed RM was extracted at a pH of 10.0 followed by precipitation at a pH of 6.0. In the study conducted by Blaicher et al., both the extraction and precipitation step were repeated three times, which could explain the high protein recovery yield. Rapeseed has a very complex protein composition, with widely different isoelectric points.

### 3.2. Protein Concentration and Mass of Sediment

The pressing method had a significant impact on the protein concentration of the precipitated protein extracts, where precipitates from hot-pressed RM and solvent-extracted RM had higher protein concentrations (79% and 72% protein on a DM basis, respectively) as compared with precipitates from cold-pressed RPC (65% protein on a DM basis) with no heat in the recovery process (Table 1).

Heat is used in the rapeseed pressing process to maximize the oil yield. Cold-pressed RPC has been pressed at lower temperatures as compared with hot-pressed RM, which results in a larger proportion of oil remaining in the starting material (the press cake); 15% oil residues for cold-pressed RPC as compared with 2% to 4% for hot-pressed RM [11]. The protein concentration in our study is reported on a dry basis of the obtained sediment to allow a proper comparison between the samples as they are otherwise diluted differently. The sediment from cold-pressed RPC had a significantly lower protein concentration as compared with sediment from hot-pressed RM, independent of the heat treatment in the recovery process. The majority of the differences could be explained by the higher oil content in the cold-pressed sediments, although co-extraction of other macromolecules (carbohydrates, fibers) was higher for cold-pressed RPC with heat treatment in the recovery process, as well as for solvent-extracted RM in the absence of heat. The sediments from cold-pressed RPC had a significantly higher oil content as compared with the hot-pressed sediments. This could be due to a higher initial oil content in the cold-pressed RPC or co-sedimentation of oil with proteins, as oleosins are efficient emulsifiers. The high oil content in the cold-pressed RPC was a surprise to the authors. It was expected that the main part of the oil would be removed in the alkali step, but the alkali extraction was performed without stirring at 4 °C which could contribute to a low reaction rate.

The total mass of the sediments differed dramatically. Precipitates from cold-pressed RPC yielded 30 ± 1 g sediment, hot-pressed RM yielded 14 ± 0.5 g, and solvent-extracted RM yielded 5.5 ± 0.3 g sediment when no heat was used in the recovery process. When heat was applied, the mass of sediment increased, although the protein concentration and overall protein yield in the sediment were significantly reduced by heat as compared with no heat in the recovery process, with the exception of protein concentration for proteins recovered from solvent-extracted RM (Table 1 and Figure 2). Heat-induced gel formation has been reported for globular rapeseed proteins upon heating, but the mechanism is not yet completely understood [37]. Ferry et al. suggested that the gel forming started with unfolding or dissociation of the protein molecules, followed by aggregation and association, and under appropriate thermodynamic conditions, formation of a gel [38]. Hermansson described a globular protein gel as an intermediate between a protein sol and precipitate where a gel could form if a proper balance between protein–solvent interactions and protein–protein interactions was achieved [39]. Schwenke et al. reported the onset of gel formation for rapeseed globulin proteins at temperatures around 72 °C which supports our findings when the heat treatment was conducted at 80 °C. Co-extracted carbohydrates, such as soluble dietary fibers, are known to also increase the water-holding capacity [40]. However, the samples were treated identically, and co-extracted carbohydrate should be in comparable levels in the two half sets until heat treatment. The difference in moisture content in the sediments between heat treated and not heat treated are probably related to gel formation of proteins, although fibers can also contribute to the elevated water holding capacity post heat treatment (Table 1).

### 3.3. Emulsifying Properties

**Figure 3:**
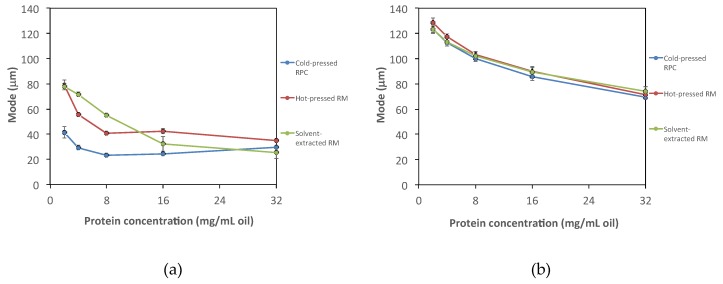
Droplet size (mode) as a function of the amount of protein per mL oil in emulsions stabilized by rapeseed protein recovered from cold-pressed RPC, hot-pressed RM, and solvent-extracted RM. (**a**) Emulsions stabilized by proteins recovered without heat and (**b**) emulsions stabilized by proteins recovered with heat. RPC, rapeseed press cake and RM, rapeseed meal.

Emulsions were prepared and analyzed to investigate possible differences in emulsifying properties of proteins extracted from rapeseed press cake or rapeseed meals with and without heat treatment in the protein recovery process. All emulsions were subjected to creaming, but no phase separation was detected. Significantly smaller emulsion droplets could be stabilized by cold-pressed rapeseed proteins, followed by hot-pressed and solvent-extracted rapeseed proteins (Figure 3a) when no heat was applied in the protein recovery process. The oil content of the protein precipitates varied depending on the oil pressing process (Table 1). When protein precipitates were added in the emulsion formulation, more rapeseed oil residues were added when protein precipitates from cold-pressed RPC were used, resulting in emulsions with slightly higher oil content emulsion as compared with emulsions stabilized by precipitates from hot-pressed RM. Protein stabilization of emulsion is a surface phenomenon, and therefore a certain amount of the cold-pressed proteins was used to cover oil droplets of rapeseed origin instead of miglyol oil droplets. Therefore, the emulsions with proteins recovered from cold-pressed RPC displayed larger emulsion particle size in our study than expected.

When heat was applied, the ability to stabilize emulsion droplets was dramatically reduced and the emulsion droplet size was similar regardless of the pressing method; non-heat treated rapeseed proteins stabilized emulsion droplets in the size interval of 20 to 80 μm (mode) depending on concentration, whereas heat-treated proteins stabilized significantly larger emulsion droplets in the interval of 80 to 130 μm (Figure 3b). The effect of heat treatment in the recovery process had a larger effect on the emulsion properties as compared with the heat applied in the previous oil pressing process. On the one hand, in the oil extraction step, the rapeseed proteins were encapsulated in the seed matrix, hence partly protected from the heat exposure. In the recovery process, on the other hand, the proteins were solubilized, and the seed matrix was separated. Proteins in this condition had no physical protection and were more sensitive to heat exposure. This could be the reason why the proteins’ ability to stabilize the water–oil interface was so dramatically reduced when heat was applied in the protein recovery process. There is also a difference between wet and dry heat, where wet heat has a higher heat transfer coefficient as compared with dry heat. In the oil pressing process, dry heat is applied, whereas in the recovery process it is wet heat, which could also contribute to the observed phenomenon.

In the literature, heat treatment of proteins has been reported to increase the emulsifying capacity for some proteins and reduce the capacity of other proteins. Leandros et al. performed a study where the effect of heat treatment on emulsifying activity was assessed for a number of different proteins. Bovine serum albumin, gluten, and whey protein were not affected by heat treatment. The emulsifying activity of soy protein was slightly improved by heating, whereas canola, pea protein, beta lactoglobulin, and casein showed decreased emulsifying activity. Leandros et al. concluded that heating did not have a uniform effect on different proteins and explained the emulsifying activity of proteins to be related to the degree of exposure of hydrophobic groups where a high degree of exposure was related to high emulsifying activity [41].

**Figure 4:**
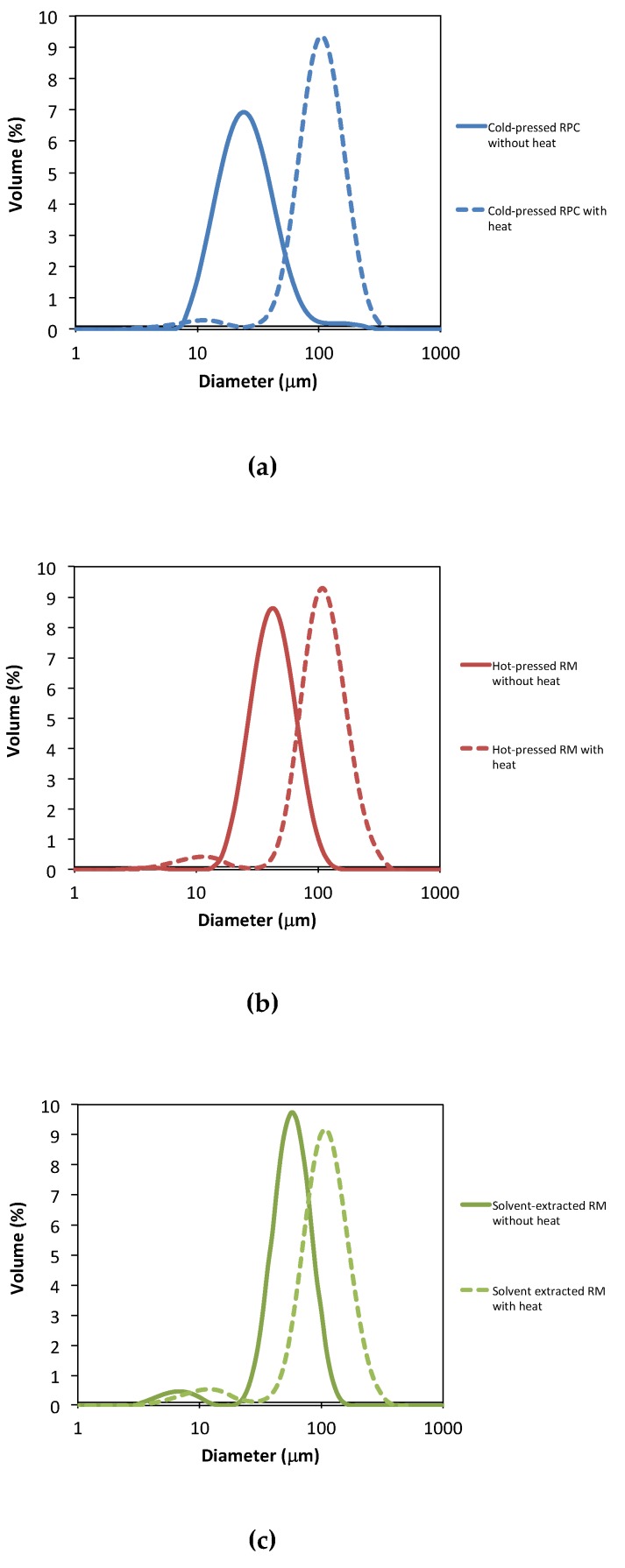
Particle size distributions of emulsion droplets stabilized by rapeseed protein recovered from (**a**) cold-pressed RPC, (**b**) hot-pressed RM, and (**c**) solvent-extracted RM. Protein concentration in the emulsions was 8 mg/mL oil. Solid lines are size distribution for emulsions droplets stabilized by rapeseed protein recovered without heat and dotted lines includes heat in the recovery process. RPC = rapeseed press cake, RM = rapeseed meal.

The particle size distributions for emulsions stabilized by protein precipitate from cold-pressed RPC and hot-pressed RM (no heat in recovery process) had a unimodal distribution, whereas all heat-treated protein precipitates and solvent-extracted protein precipitates had a bimodal distribution with a smaller peak around 10 μm (Figure 4). The homogenization device used in our study is limited to produce oil droplets above 10 μm. In a preliminary study conducted by our lab, rapeseed sediment was dispersed in a phosphate buffer and was mixed at the same conditions as the emulsions were prepared. The dispersion was added dropwise to the sample container of the particle sizer and displayed a peak in the size region of 7 μm to 9 μm. Therefore, we conclude that the observed peak around 10 μm is not oil droplets but proteins that could not attach to the oil–water interface and therefore was found in the continuous phase of the emulsion.

The results in our study are in line with findings reported by Khattab and Artnfield [42] where heat treatment was found to significantly reduce the emulsifying properties of the rapeseed proteins. Khattab and colleagues also reported that moist heat treatment such as boiling or industrial desolventizing processes was found to further reduce the proteins’ ability to stabilize emulsions as compared with dry heat treatment, such as roasting, which is significant for hot pressing [42]. However, there were differences in the procedure, i.e., Khattab et al. stabilized emulsions by crude rapeseed meal defatted by hexane, whereas emulsions in our study were stabilized by precipitated rapeseed proteins without the use of solvents. He et al. reported evidence for altered secondary and tertiary protein structure after heat treatment (60, 80, and 100 °C for 15 min) of rapeseed protein slurry, where heat treatment induced protein aggregation, resulting in an increased hydrophobicity [43]. By exposure to heat during the protein recovery process, the balance between exposed hydrophobic and hydrophilic regions seemed to be altered, resulting in reduced emulsifying properties. In contrast to our study, Flores-Jiménez et al. showed that partial denaturation and formation of a more disordered structure increased the proteins’ emulsifying activity, i.e., the proteins’ ability to absorb to the oil–water interface [44]. High intensity ultrasound was used to create the change in structure and the raw material was canola meal, which could also contribute to the different outcome.

**Figure 5:**
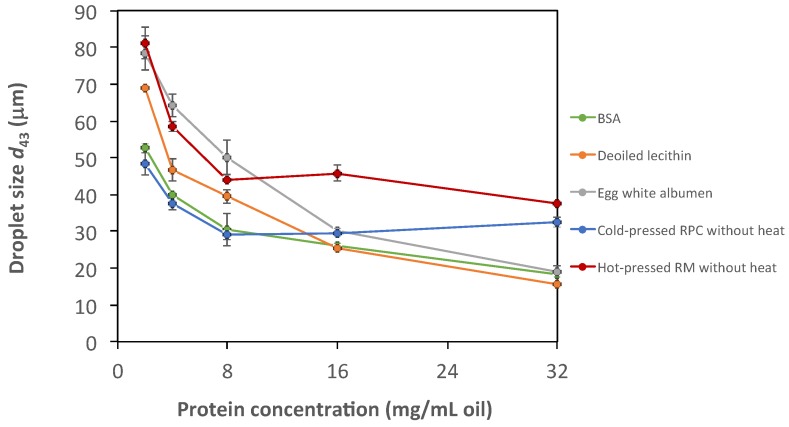
Emulsion droplet size (*d*_43_) for emulsions stabilized by recovered rapeseed proteins and commercial emulsifying agents. RPC, rapeseed press cake and RM, rapeseed meal.

Proteins from cold-pressed RPC without heat in the recovery process stabilized emulsion droplets at the same or smaller size as commercial emulsifying agents such as bovine serum albumin, deoiled soy lecithin, and egg white albumen for concentrations up to 16 mg protein/mL oil (Figure 5). All emulsions were subjected to creaming, but no phase separation was detected. The homogenizer used in our study produced coarse emulsions and the droplet size was not small enough to prevent some destabilization processes such as creaming. Therefore, the system is used as a model to compare different emulsifiers, rather than exploring the smallest emulsion droplet size that can be produced. Proteins from hot-pressed RM had a similar ability to stabilize emulsions as egg white albumin at least at low protein concentrations, but at higher protein concentration the ability to stabilize emulsion droplets was reduced as compared with the commercial emulsifiers investigated. The protein BSA from animal origin and proteins recovered from cold-pressed RPC (without heat in the recovery process) were found to have equal emulsifying capacity in lower concentrations. The slight increase in emulsion droplet size at higher concentrations of rapeseed extracts is not fully known. However, one explanation could be a degree of flocculation. The rapeseed protein extract was slightly acidic and emulsions varied between 6.7 (2 mg protein/mL oil) to 5.7 (32 mg protein/mL oil) which could affect solubility and flocculation. Since a larger mass of extract was also added for the higher protein concentrations, higher amounts of non-protein compounds were added as well. These compounds could cause depletion flocculation in the system with larger emulsion droplets as consequence.

## 4. Conclusions

Proteins were recovered from industrially cold-pressed rapeseed press cake (RPC) with a higher protein recovery yield as compared with hot-pressed rapeseed meal (RM) and solvent-extracted RM. Proteins from cold-pressed RPC had better emulsifying properties as compared with hot-pressed and solvent-extracted rapeseed proteins and were in the same range as commercial emulsifying agents in concentrations up to 16 mg protein/mL oil. When heat was applied in the recovery process, the protein recovery yield was slightly improved for solvent-extracted RM, no difference was found for hot-pressed RM, and a reduced yield was found for cold-pressed RPC. The ability to stabilize emulsions was significantly reduced when heat was applied in the protein recovery process, and the pressing method no longer affected the results. Our study suggests that industrially cold-pressed RPC without heat in the recovery process could be a successful strategy for an efficient recovery of rapeseed protein with good emulsifying properties.

## Figures and Tables

**Table 1:** Characterization of isolated rapeseed protein from cold-pressed RPC, hot-pressed, and solvent-extracted RM with and without heat in the recovery process. DM basis is dry matter basis.

Pressing Process	Heat in Isolation Process	Wet Mass of Sediment (g)	Moisture Content of Sediment (%)	Protein Concentration of Sediment (%) DM Basis	Oil Content of Sediment (%) DM Basis
Cold-pressed RPC	No	30 ± 1	68 ± 0.9	65 ± 2	20 ± 0.4
Hot-pressed RM	No	14 ± 0.5	59 ± 0.9	79 ± 3	7.9 ± 0.8
Solvent-extracted RM	No	5.5 ± 0.3	80 ± 1	72 ± 18	2.4 ± 0.3
Cold-pressed RPC	Yes	43 ± 1	82 ± 1	44 ± 4	19 ± 0.2
Hot-pressed RM	Yes	31 ± 2	80 ± 2	61 ± 10	9.5 ± 0.4
Solvent-extracted RM	Yes	11 ± 0.4	89 ± 1	88 ± 1	1.5 ± 0.3

RPC is rapeseed press cake and RM is rapeseed meal. Protein recovery was performed in triplicate and each sample was analyzed in triplicate. Data are given as mean ± standard deviation.
